# Evolution of pathogen virulence: Studying the complex interplay of pathogen interactions, virulence and transmission helps us understand how they evolve and spread

**DOI:** 10.15252/embr.202357611

**Published:** 2023-07-19

**Authors:** Sunetra Gupta

**Affiliations:** ^1^ Department of Biology University of Oxford Oxford UK

**Keywords:** Evolution & Ecology, Microbiology, Virology & Host Pathogen Interaction

## Abstract

Navigating the intricate interplay of competitive and co‐operative interactions and the complex relationship between virulence and transmission pose challenges for our understanding of how pathogens evolve and spread.

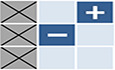

During the time that we have watched the dynamics of SARS‐CoV‐2 unfold, there has been a tacit acceptance of the notion that all pathogens evolve towards higher transmissibility and lower virulence. The lower fatality rates of the Omicron variant have thus been ascribed to a diminution in its virulence, and its high attack rates have been put down to a very rapid increase in its transmissibility, often represented by the basic reproduction number *R*_0_ – a construct which reflects a pathogen's fundamental ability to spread from person to person within a particular setting.

Do these assumptions have a basis in our current understanding of how pathogens evolve?

## Pathogen spread and herd immunity

**Figure 1:**
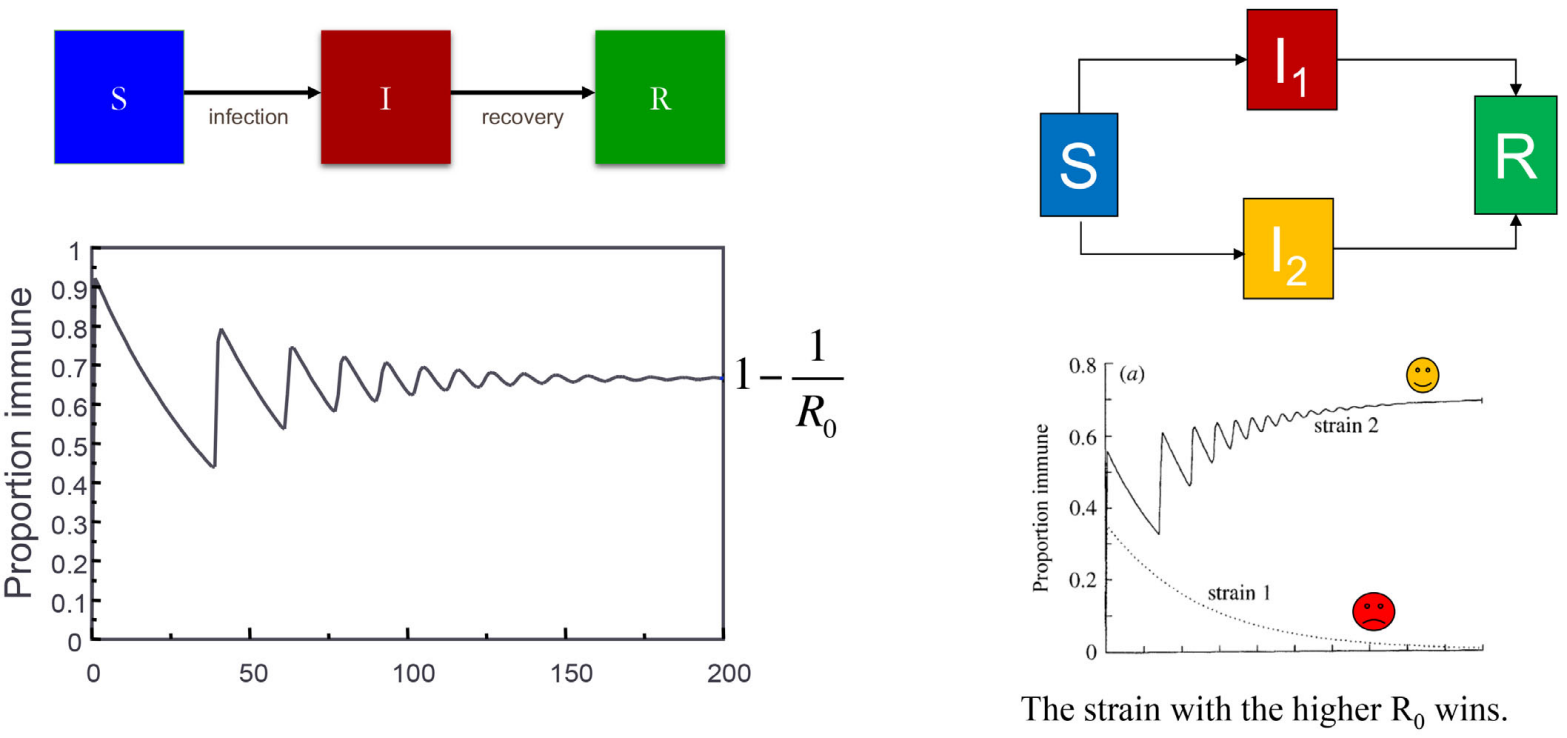
Strains with higher transmissibility will outcompete others in the absence of immune evasion The dynamics of an epidemic are shown on the right in terms how the proportion of a population that is immune to a pathogen changes over time. This is based on a simple model where individuals progress from a susceptible compartment (S) to an infectious compartment (I) and eventually to a recovered compartment (R). The proportion immune will always converge to a value 1‐1/R_o_, where R_o_ is a measure of the intrinsic transmissibility of the pathogen. The figure on the left shows how the system behaves if there are two different strains of the pathogen (red and yellow); in this situation the model has to be extended to accommodate the possibility of being infected by either the yellow or red strain, and eventually the strain with the higher R_o_ will outcompete the other.

To answer this question, we need to consider how pathogens spread when they are introduced into a population that has no previous exposure to it. We know, from the fundamental principles of epidemiological modelling (Anderson & May, 1991), that a pathogen will spread, provided *R*_0_ is larger than one – in simple terms, that an infected individual in a totally susceptible population is, on average, able to infect at least one other person. We also know that, as the pathogen spreads, it will slow itself down by reducing the availability of susceptible individuals. Eventually, it reaches an equilibrium state where the proportion of immune individuals hovers around what is known as the herd immunity threshold (generally given by 1−1/*R*_0_) with regular fluctuations in numbers infected depending on seasonality and other factors (Fig 1). Ecologists will be familiar with the concept of a carrying capacity – and that is precisely what the herd immunity threshold represents. Whether or not immunity is lifelong or lost rapidly does not affect the movement of an invading pathogen towards this inevitable endemic equilibrium (Gupta, 2022).

While susceptible hosts are in abundance (similar to lots of cake being available), different variants of the pathogen may happily coexist. But when hosts become resistant after infection (when most of the cake has been eaten), these same variants will be in strong competition (fighting for the last slice of cake) and the variant with the highest *R*_0_ will win (Fig 1). The winning variant does not need to be much better at infecting others; it just needs to be good enough to win the race. Given this well‐established epidemiological principle (Gupta *et al*, 1994a), it would be unwise to infer that the *R*_0_ increased by leaps and bounds during the recent COVID pandemic. A simpler explanation is that the winning variants acquired a slight transmission advantage and consequently took over.

The winning variant does not need to be much better at infecting others; it just needs to be good enough to win the race.

Would such a winning variant more likely be less virulent than that which is has displaced? It is tempting to assume that a lower mortality rate would always increase transmissibility, but this is complicated by the fact that the same traits that favour transmission may also enhance virulence. For example, a variant that grows faster may well cause a more severe disease, but the higher levels of viraemia may also enhance transmission. It is thus widely accepted now (Geoghegan & Holmes, 2018) that pathogens will evolve to optimise virulence rather than minimising the likelihood of killing the host. A classic example of evolution towards optimal virulence is the trajectory of the myxoma virus in Australia where it was introduced in 1950 to control the rabbit population (Kerr *et al*, 2012). The original strain employed had a very high fatality rate in rabbits, but it was soon outcompeted by descendent variants which were not quite so virulent. Although the virus is capable of becoming even milder, we did not observe such continued attenuation of virulence, simply because the very mild forms do not cause the sores on the rabbit from which the virus gets picked up by flies and transmitted to other rabbits. The strains that won the race were those which did not kill as many bunnies but did cause enough symptoms to promote their spread.

It is tempting to assume that a lower mortality rate would always increase transmissibility, but this is complicated by the fact that the same traits that favour transmission may also enhance virulence.

It is therefore simply not true that all pathogens evolve towards low virulence, and there is no evolutionary argument to support the idea that Omicron won the race by becoming milder. A more parsimonious explanation for its apparently milder profile would be that most of the vulnerable population had already acquired immunity to severe disease through natural exposure or vaccination by the time Omicron came to dominate (Saad‐Roy *et al*, 2020; Lavine *et al*, 2021).

## Switching diet

Another important point to consider is that an advantage Omicron had over the other variants is that it was no longer competing for exactly the same resources. To continue with the cake analogy, you might say that part of Omicron's diet had switched to apple pie. It had acquired a significant number of mutations compared to the other variants which meant the immune responses established by these other variants were no longer as effective. As a result, Omicron was more readily able to infect people who had still had immunity against the Delta variant, for example. This is a major reason for its emergence, rather than any sharp increase in transmissibility or attenuation of virulence. Indeed, phylogenetic studies indicate that Omicron had been hiding in the wings all along, waiting to make an entrance when the conditions were right. That it was not able to outcompete the other variants earlier suggests that its transmissibility might actually be lower but it made up for it through properties of immune evasion.

… phylogenetic studies indicate that Omicron had been hiding in the wings all along, waiting to make an entrance when the conditions were right.

The evolution of pathogen virulence can thus only be understood in the context of immunological interactions between variants, as well as the complex relationship between virulence and transmission. Infection‐blocking immunity creates competition between variants, leading to victory or competitive exclusion. However, if a variant acquires the ability to avoid “cross‐immunity”, it can succeed even if it is less transmissible (Gupta *et al*, 1994a). Many pathogens, such as the malaria parasite, exist as a set of co‐circulating strains because each has evolved to avoid “cross‐immunity” with the others (Gupta *et al*, 1994b). This is achieved through a high degree of diversity among the principal targets of protective immunity. In cake analogy terms, the parasite population has differentiated into strains each of which primarily consumes a different article: cake, pie, trifle, meringue. Because they are no longer competing with each other, they are able to coexist even with vast differences between them in transmissibility and virulence (Jensen *et al*, 2019). For malaria, as well as many similarly diverse bacterial pathogens, only certain strains or variants appear to have the capacity to cause severe disease.

Interactions between variants do not always have to occur at the level of blocking infection or transmission altogether. Previous exposure to other strains, or even other related pathogens, can provide immunity against severe disease. It is very likely that the 2009 swine influenza pandemic did not cause the same devastation as its predecessor in 1918 because repeated exposure to influenza within the population created durable protection against severe disease. Particularly strong immune responses to the 2009 strain were noted among those who had been alive during the period that Spanish flu arrived and subsequently circulated (Miller *et al*, 2010) – this can be explained by the high degree of similarity between the strains in the “head” region of its haemagglutinin protein, which is the primary target of our neutralising antibody responses.

The evolution of pathogen virulence can thus only be understood in the context of immunological interactions between variants, as well as the complex relationship between virulence and transmission.

SARS‐CoV‐2 emerged as a novel pathogen against a background of four circulating coronaviruses, as well as a history of previous exposure to SARS‐CoV‐1 and the MERS virus in some parts of the world. It is now well‐established that pre‐existing immune responses to these other strains were able to mitigate the clinical effects of SARS‐CoV‐2 (Kundu *et al*, 2022). Recent exposure to a related coronavirus may even have prevented infection with SARS‐CoV‐2 in some individuals during the first wave. So, although it might not seem that way, the virus was already facing an uphill struggle to establish itself and spread through the human population. Indeed, this may be why we do not see regular incursions of a new coronavirus from the various species from which they could spill over into human hosts – it is hard for them to breach the protective barrier conferred by our constant exposure to other coronaviruses.

SARS‐CoV‐2 was, however, evidently able to cross this barrier, exhibiting patterns that closely conform to the expected behaviour of a coronavirus similar to those currently circulating in humans. The salient features of this family of viruses are that immunity against infection is short‐lived, while immunity against severe disease is long‐lived and therefore typically acquired upon first infection; furthermore, there is a seasonal pattern to transmissibility. Putting these ingredients into a mathematical model (introducing seasonality into what is known as an SIRS model – where hosts go from being susceptible to infected to recovered and then back to being susceptible) yields a dynamic picture that fits well with our observations of the spread of SARS‐CoV‐2 and readily explains the change in mortality associated with the emergence of Omicron, as already discussed (Gupta, 2022).

## The effects of vaccination on virulence

Given the strong likelihood that a single exposure to SARS‐CoV‐2 would lead to lifelong protection from severe disease, the correct strategy was clearly to develop as rapidly as possible a vaccine for those at significant risk of severe disease. Such vaccines were produced and rolled out to populations both at risk and not at risk of severe disease under the assumption that they would durably block infection which – not surprisingly – they did not. How did this affect the evolution of pathogen virulence? It is possible that the transient re‐enforcement of infection‐blocking immunity against the earlier strains increased the differential advantage of the Omicron variant but this could have resulted in an increase in intrinsic virulence as much as a decline. As I have already explained, the most plausible outcome is that there was no change at all in intrinsic virulence and the fall in hospitalisable illness occurred because most people had already acquired immunity against severe disease. Vaccines would thus have played a role in altering virulence by reducing the proportion vulnerable to severe disease rather than by inducing a change in the virus population itself.

… the correct strategy was clearly to develop as rapidly as possible a vaccine for those at significant risk of severe disease.

In more general terms, vaccinating a population to prevent severe disease can theoretically cause a shift towards higher virulence as it shields the more virulent variants from the negative effects of high virulence – death of host – and thus makes them more competitive. This was proposed by Andrew Read and colleagues several years ago and potentially explains the increase in virulence of Marek's Disease among poultry following mass vaccination (Read *et al*, 2015). As we move into an era where more and more vaccines are being produced to specifically target severe disease rather than infection, we may wish to keep a weather eye out for such shifts in virulence although we will be safe from their harms provided everyone at risk is vaccinated.

**Figure 2:**
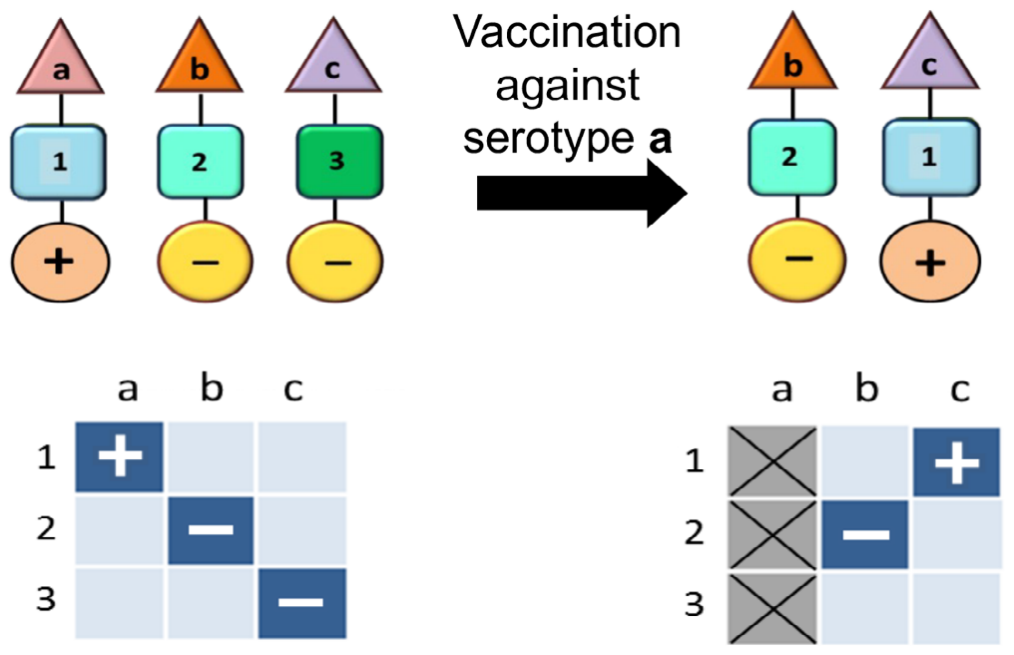
Pneumococcal protein conjugate vaccines can alter the genomic profile of non‐vaccine serotypes, potentially leading to an increase in their transmissibility and virulence Pneumococcal and other pathogenic bacterial populations are commonly characterised by non‐overlapping associations between antigenic (shown by the triangle) and metabolic types (shown by the square) as well as virulence factors (shown by the circle). In this schematic the antigenic types (a, b, c) are immunologically distinct (i.e. no cross‐immunity), while the metabolic types (Anderson & May, 1991; Gupta *et al*, 1994a; Gupta, 2022) are ecologically distinct (i.e. they utilise completely different resources and receptors). Certain metabolic types may be associated with increased virulence (+). If the metabolic types differ in their fitness, vaccination against a particular serotype (in this example, serotype **a**) can induce a rearrangement in the population structure whereby the fitter metabolic type (in this example, serotype **a**) appears to shift to a different serotype, also causing it to increase in virulence.

These considerations mainly pertain to pathogens which are not differentiated into multiple strains, each with different levels of virulence. A vaccination strategy that can be employed against multi‐strain pathogens is to only target the virulent strains. This is precisely the thinking behind the vaccines to reduce the colossal death rates from *Streptococcus pneumoniae*. However, this can lead to a population‐wide genomic rearrangement whereby previously non‐virulent strains acquire the same characteristics as the disease‐causing strains against which the vaccine has been developed (Watkins *et al*, 2015). This is because the association between virulence factors and immunological determinants is essentially modular – thus, virulence factors will inevitably be accommodated by the strains/variants which are not under assault when the original strains which harboured them are eliminated through vaccination (Fig 2).

## The efficiency of NPIs

Non‐pharmaceutical interventions (NPIs) were widely implemented during the COVID pandemic in effort to curb the spread. It is extremely difficult to slow the spread of a pathogen in its epidemic phase through these methods as it has a large susceptible population at its disposal (lots of cake left, so taking away a slice does not have much effect). Consequently, to which extent NPIs had effect on the spread of SARS‐CoV‐2 other than in a few exceptional circumstances is still unclear.

However, NPIs can have a profound effect on endemic diseases – very little cake around, so taking away a slice has a large effect – and, indeed, we witnessed a temporary disappearance of many common pathogens such as influenza and RSV. When they returned after the hiatus, some of the symptoms they caused in children were clearly more severe. It is unlikely however that the pathogens themselves had gained in virulence; what is more plausible is that the delay in exposure among young children had rendered them more vulnerable either because they had lost the maternal antibodies that would have given protection from severe outcomes or because the severity of disease increases with age.

Several different viruses rebounding at the same time also increases the chance of coinfection, which may lead to worse outcomes.

Several different viruses rebounding at the same time also increases the chance of coinfection, which may lead to worse outcomes. Two independent studies (Ho *et al*, 2023) found that acute severe hepatitis in children was linked to the presence of adeno‐associated virus 2 (AAV2) alongside adenovirus in spring 2022. AAV2 is a “satellite” virus as it relies on a “helper” virus such as adenovirus or herpesvirus in order to replicate, and was present in 96% of cases of hepatitis in both studies. The hepatitis D virus (HDV) is another example of a “satellite” virus which steals the surface proteins of hepatitis B virus (HBV) to complete its lifecycle. In this case too, the combination of HDV and HBV is significantly more dangerous than HBV on its own. NPIs can disrupt the delicate ecology of our relationship with pathogens leading to short‐term increases in their apparent virulence but we can expect them to settle back eventually into their normal patterns.

## A matter of competition and cooperation

The evolution of pathogen virulence is thus determined by a complex combination of competitive and co‐operative interactions between variants of the same pathogen as well as between related pathogens. Competition is mainly mediated by infection‐blocking immunity, although many pathogens also compete directly for resources such as metabolites or host receptors. Under strong competition, the more transmissible variants will displace the others; when the immune fraction within a population is close to the herd immunity threshold, only a slight transmission advantage can cause a variant to take over rapidly. Such a variant is not necessarily attenuated in virulence, as – contrary to popular belief – reducing virulence does not always increase transmissibility; instead, maximum transmissibility in often associated with an intermediate “optimum” level of virulence.

… reducing virulence does not always increase transmissibility; instead, maximum transmissibility in often associated with an intermediate “optimum” level of virulence.

Pathogen variants can avoid competition by differentiating at targets of immunity or by consuming different resources. In this case, many different variants may co‐exist provided they partition their resources by avoiding cross‐immunity or utilising different metabolites and receptors. These variants may differ quite widely in their levels of virulence. However, it also becoming increasingly clear that some of the shared immune responses between these variants or “strains” can offer significant protection against severe disease upon reinfection with a different strain. This principle may extend to all wider networks of related pathogens, and could be the reason why we are no longer prey to devastating “virgin soil” pandemics in this age of high international mobility. The same bugs which would have wiped us out a hundred years ago have now been rendered less harmful by our extensive and continuous exposure to related pathogens.

### Disclosure and competing interests statement

The author declares that she has no conflict of interest.

## Supporting information


