# Supplementary material for: Evolution of pathogen virulence: Studying the complex interplay of pathogen interactions, virulence and transmission helps us understand how they evolve and spread
Source: EMBO Rep. 2023 Jul 19;24(8):e57611. doi: 10.15252/embr.202357611 (PMC10398641; doi:10.15252/embr.202357611)
Supplement: Supplementary file 1 [file EMBR-24-e57611-s001.pdf]

EMBO reports

Evolution of pathogen virulence

Sunetra Gupta
DOI: 10.15252/embr.202357611

Corresponding author(s): Sunetra Gupta (sunetra.gupta@biology.ox.ac.uk)

Review Timeline:

Submission Date:	9th Jun 23
Accepted:	5th Jul 23

Editor: Holger Breithaupt

Transaction Report:

(Note: With the exception of the correction of typographical or spelling errors that could be a source of ambiguity, letters and reports are not edited. Depending on transfer agreements, referee reports obtained elsewhere may or may not be included in this compilation. Referee reports are anonymous unless the Referee chooses to sign their reports.)
